# Recruitment of reviewers is becoming harder at some journals: a test of the influence of reviewer fatigue at six journals in ecology and evolution

**DOI:** 10.1186/s41073-017-0027-x

**Published:** 2017-03-08

**Authors:** Charles W. Fox, Arianne Y. K. Albert, Timothy H. Vines

**Affiliations:** 1 Department of Entomology, University of Kentucky, Lexington, KY 40546-0091 USA; 2 Women’s Health Research Institute, BC Women’s Hospital and Health Centre, Vancouver, British Columbia V6H 3N1 Canada; 3 Axios Review Editorial Office, 4521 John Street, Vancouver, British Columbia V5V 3X3 Canada

**Keywords:** Peer review, Reviewers, Reviewer fatigue, Scholarly journals

## Abstract

**Background:**

It is commonly reported by editors that it has become harder to recruit reviewers for peer review and that this is because individuals are being asked to review too often and are experiencing reviewer fatigue. However, evidence supporting these arguments is largely anecdotal.

**Main body:**

We examine responses of individuals to review invitations for six journals in ecology and evolution. The proportion of invitations that lead to a submitted review has been decreasing steadily over 13 years (2003–2015) for four of the six journals examined, with a cumulative effect that has been quite substantial (average decline from 56% of review invitations generating a review in 2003 to just 37% in 2015). The likelihood that an invitee agrees to review declines significantly with the number of invitations they receive in a year. However, the average number of invitations being sent to prospective reviewers and the proportion of individuals being invited more than once per year has not changed much over these 13 years, despite substantial increases in the total number of review invitations being sent by these journals—the reviewer base has expanded concomitant with this growth in review requests.

**Conclusions:**

The proportion of review invitations that lead to a review being submitted has been declining steadily for four of the six journals examined here, but reviewer fatigue is not likely the primary explanation for this decline.

The process of peer review serves two primary purposes—reviewers advise editors on which papers to include in their journal and provide constructive feedback to authors to improve the quality of their research and paper. Success of the peer review system relies on the willingness of the research community to review manuscripts, which is usually unpaid. The research community depends on individuals who volunteer their time for peer review, but few direct rewards exist at the individual level to encourage reviewing [1]. Given the tremendous growth in submissions that many journals are experiencing (e.g., [2]), it is unsurprising that many editors have reported that it is getting harder to recruit reviewers for manuscripts [3]. There is a common perception that reviewers are increasingly being asked to review too often (certainly more than in the past) and are thus experiencing reviewer fatigue ([4, 5]; but see [6]), but there is little published evidence to support this.

In a recent analysis of peer review at five ecology journals, Albert et al. [7] examined how often invitations sent to prospective reviewers lead to a submitted review and tested whether the number of review invitations prospective reviewers receive has been increasing over a 7–8-year period. They found that the proportion of review requests that lead to a completed review declined over this period for four of the journals, but the decline was not substantial, and there was no evidence of a decline at a fifth journal. They also found that the number of review requests sent to an average reviewer had not increased substantially over the period of their study.

Here, we extend the analyses of Albert et al. [7] to additional years (13 years instead of 8) and two additional high impact factor journals (*Evolution* and *Methods in Ecology and Evolution*; 2015 impact factors are >4.0 for all six journals). Also, unknown to Albert et al. [7], the dataset available online for the four journals of the British Ecological Society [8, 9] contains errors that influenced some of their results (but not their main conclusions). We thus analyze a newly compiled dataset for these journals and present updated/corrected figures.

Averaged across journals, we see a significant decline over the 13 years (2003–2015) in the frequency of review invitations that led to a review being submitted (cyan line in Fig. 1). However, this decline varied quite substantially among journals. For four journals (*Functional Ecology*, *Journal of Animal Ecology*, *Journal of Applied Ecology*, and *Journal of Ecology*), the decline is large and fairly consistent over the entire 13 years (logistic regression, *Response* = *Year*, with *Year* as a continuous variable; *χ* ^*2*^_*1*_ *>* 299.0, *P* < 0.001 for each), from an average (across journals) of 56% of review invitations generating a review in 2003 to just 37% in 2015 (cyan lines in Fig. 1). In contrast, there has been no decline for *Evolution* or *Methods in Ecology and Evolution*, although we have fewer years of data for those journals. The slopes of the lines, especially for *Evolution*, clearly differ from those of the other journals. The results for these two journals match the lack of trend found for *Molecular Ecology* in Albert et al. [7].

**Fig. 1:**
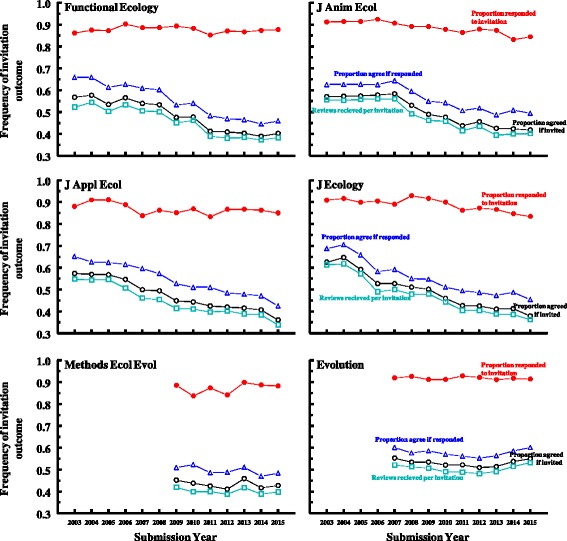
Declines in reviewer recruitment success for standard research papers at six journals in the fields of ecology and evolution. The lines are the proportion of invited reviewers who responded to the invitation email (red line, filled circles), the proportion of respondees who agreed to review (blue line, triangles), the proportion of all invited reviewers who agreed to review (black line, open circles), and the proportion of all invitations that generated a submitted review (cyan line, squares). Standard papers include traditional research papers and excludes commentaries, perspectives, brief communications, and any other manuscript type not designated “original article” (*Evolution*), “research article” (*Methods in Ecol Evol*), or “standard paper” (the remaining journals). This also excludes revisions and, for *Evolution*, resubmissions of previously rejected papers. Analyses: Logistic regression, *Response = Year* + *Journal* + Year*Journal interaction, with *Year* as a continuous variable. (A) Proportion of invitees responding to invitation (red line, filled circles): *Year: χ* ^*2*^_*1*_ = 18.7, *P <* 0.001, *Journal: χ* ^*2*^_*1*_ = 109.2, *P* < 0.001, *Interaction: χ* ^*2*^_*1*_ = 109.2, *P* < 0.001; (B) proportion of respondees agreeing to review (blue line, triangles): *Year: χ* ^*2*^_*1*_ = 347.1, *P* < 0.001, *Journal: χ* ^*2*^_*1*_ = 150.7, *P* < 0.001, *Interaction: χ* ^*2*^_*1*_ = 151.1, *P* < 0.001; (C) proportion of invitees agreeing to review (black line, open circles), *Year: χ* ^*2*^_*1*_ = 352.6, *P* < 0.001, *Journal: χ* ^*2*^_*1*_ = 162.2, *P* < 0.001, *Interaction: χ* ^*2*^_*1*_ = 162.7, *P* < 0.001; (D) proportion of all invitations generating a review (cyan line, squares): *Year: χ* ^*2*^_*1*_ = 313.4, *P* < 0.001, *Journal: χ* ^*2*^_*1*_ = 171.0, *P* < 0.001, *Interaction: χ* ^*2*^_*1*_ = 171.5, *P* < 0.001

The decline in reviews received per invitation sent for these four journals (*Functional Ecology*, *Journal of Animal Ecology*, *Journal of Applied Ecology*, and *Journal of Ecology*) is driven primarily by a decline in the proportion of invitees agreeing to review when they respond to the invitation (blue line in Fig. 1); the decline in the proportion of respondents who agree to review was significant for all journals except *Evolution* (Fig. 1; *χ* ^*2*^_*1*_ *>* 3.9, *P* < 0.05 for all except *Evolution*, for which *χ* ^*2*^_*1*_ *=* 0.0, *P* = 0.99). For the four journals with the steepest declines, we see a drop from 66% of respondents agreeing to review in 2003 to just 46% agreeing in 2015 (averaged across journals). Also contributing is a small decline in the proportion of invitees who responded to the invitation email (red line in Fig. 1), though this also varied among journals. The proportion of invitees who responded declined significantly over time for *J Animal Ecology*, *J Applied Ecology*, and *J Ecology* (*χ* ^*2*^_*1*_ *>* 29.2, *P* < 0.001) but not the others (for which *χ* ^*2*^_*1*_ *<* 2.1, *P* > 0.15) (response rates actually increase slightly but significantly over the few years for which we have data for *Methods in Ecology and Evolution*).

Albert et al. [7] also asked whether reviewer fatigue might explain a decline in the willingness of people to review for journals. They found no evidence that the number of invitations sent to specific individuals has increased over time at any of the journals they examined. This remains true in our expanded dataset. The average number of invitations sent to each invitee varied substantially among journals (significant Year*Journal interaction; Fig. 2a) but, despite substantial increases in the total number of review invitations sent by editors cumulative across all invitees (Fig. 2b), there was no consistent increase over time in the average number sent to each individual invitee. There was a decline over time for *J Animal Ecology* and *J Applied Ecology*, an increase for *J Ecology*, and no significant directional change over time for the other journals. Because five of the journals examined here use a common reviewer database, we can also ask whether the average number of invitations sent to individuals across all of these five journals increased over time (brown line, solid circles in Fig. 2a), but we see that the pattern does not change, and there is no evidence for a consistent increase over time.

**Fig. 2:**
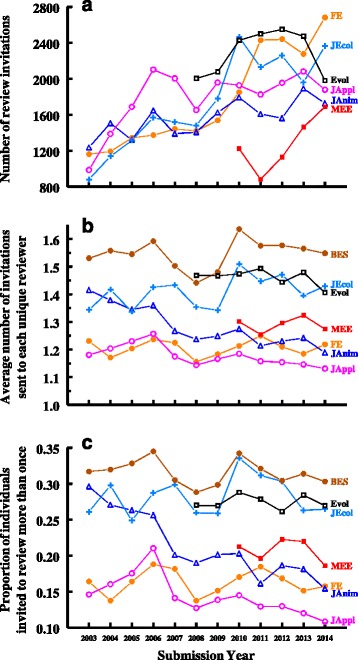
The **a** total number of reviewers invited, **b** average number of invitations sent to each unique reviewer, and **c** proportion of reviewers invited more than one time within a given year, for “standard papers” (defined as in Fig. 1) submitted to six journals of ecology and evolution. The five journals published by the British Ecological Society (*Functional Ecology, Journal of Animal Ecology, Journal of Applied Ecology, Journal of Ecology, and Methods in Ecology and Evolution*) share a common reviewer database; the line labeled BES (top brown line, filled circles) is the average number of invitations sent to each unique reviewer across all five BES journals. These estimates (all lines in b and c) likely underestimate the true number of invitations sent to each researcher due to duplicate accounts in *ScholarOne Manuscripts*, though this error should be very small. We exclude 2015 (all journals), 2009 for *Methods in Ecology and Evolution* and 2007 for *Evolution* because we have data for only part of those year and thus numbers of invitations are not comparable with other years. Analysis for (B): Analysis of covariance, log(*NumberOfTimesInvited*) = *Year* + *Journal* + Year*Journal interaction, with year as a covariate; *Year*: F_1,81301_ = 4.64, *P* = 0.03; *Journal*: F_5,81301_ = 27.0, *P* < 0.001; Year*Journal: F_1,81301_ = 27.1, *P* < 0.001. The means here differ slightly from those in Albert et al. (2016) for the journals / years in common between studies because (A) duplicate accounts are merged as found, reducing the number of unique reviewers and thus increasing our means per individual reviewer relative to theirs, and (B) in early years of the dataset their dataset double counts review invitations for reviewers of papers that were invited for revision, inflating their estimates for some journals and years (this is especially evident for *Functional Ecology* and *Journal of Applied Ecology*; see text for details)

However, the means and the patterns in Fig. 2b are difficult to interpret because most individuals are invited just once in any given year (i.e., the median invitations per individual is just 1 for all journals in all years). We thus examined the proportion of individuals invited more than once in any given year. As in the above analysis, there was a general decline over time for *J Animal Ecology* and *J Applied Ecology*, but no change over time for the remaining journals. This is likely because, despite the need to invite substantially increasing numbers of individuals over time at each of these journals, the journals are broadening their reviewer populations rather than increasing the burden per individual reviewer. *J Applied Ecology* and *Functional Ecology* have the most diverse reviewer pools (inviting only 14.4 and 16.3% of individuals more than once per year, averaged over years), and *J Ecology* and *Evolution* have the least diverse reviewer pools (28.3 and 27.5% of their invitees are invited more than once). On average, individual journals invite only 21% of individuals more than once per year, 5.5% more than twice, 1.2% more than three times, and 0.6% more than four times. Across the five journals of the British Ecological Society, which share a common reviewer database (all journals presented here except *Evolution*), most reviewers are invited only one time (across all journals) in any given year—only 32% of reviewers are invited more than once within any calendar year, 12.8% more than twice, 5.7% more than three times, and 2.6% more than four times.

Despite the lack of an overall increase in the average number of invitations sent to each unique reviewer and the lack of a change over time in the proportion of reviewers invited more than once, we do find evidence of reviewer fatigue at the individual reviewer level—the probability that a reviewer agreed to review for a journal was negatively correlated with the number of review invitations they received from that journal that year (Fig. 3). Individuals invited just once in a calendar year agreed to review 56% of the time, on average across years and journals, whereas individuals invited 6 times in a year agreed just 40% of the time. This indicates that editors are not generally repeat-inviting reviewers who are more likely to agree to review. It’s also consistent with the hypothesis that individuals experiencing reviewer fatigue are more likely to decline review invitations. Alternatively, journals may intentionally avoid overusing their reviewers by not repeat-inviting individuals who have recently agreed to review (generating a negative relationship between number of invitations and agreement probability). *Functional Ecology*, for example, avoids inviting individuals who have reviewed for the journal too recently (within the past 2–3 months) as long as alternative individuals with similar expertise are available to invite. Regardless of the explanation, the negative relationship between the probability that an individual agrees to review and number of invitations does not explain the patterns in Fig. 1; even after deleting all individuals who received more than one invitation from an individual journal in a single calendar year, the decline in the proportion of respondents agreeing to review remains significant (and similar in magnitude) for the same journals as described above.

**Fig. 3:**
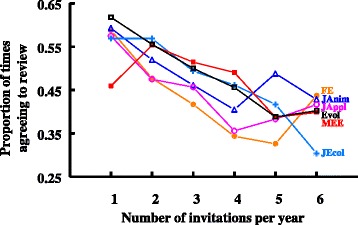
Reviewers who are invited more times within a single calendar year are more likely to decline to review. The Y-axis is the proportion of times an invitee agreed to review, averaged first across unique individuals within a calendar year and then across years within each journal. Invitations greater than 6 per year (x-axis) are excluded (treated as outliers) because sample sizes are very low and the patterns become uninterpretable. The figure and analysis excludes 2015 (all journals), 2009 for *Methods in Ecology and Evolution* and *2007* for *Evolution* because we have data for only part of those year and thus numbers of invitations are not comparable with other years. This analysis also excludes reviewers who did not respond to the email invitation; the overwhelming majority of non-responses were unique invitations, suggesting incorrect contact information. Analysis: Analysis of covariance, with each journal contributing one data point per reviewer invitation count per year; model: *ProportionAgreed = Year + Journal + TimesInvited* + 2-way interactions, with *TimesInvited* as a continuous variable; *Year: F*_11,253_ = 1.13, *P* = 0.34; *Journal: F*_5,253_ = 0.71, P = 0.62, *TimesInvited: F*_1,253_ = 54.2, *P* < 0.001, Year*Journal: F_44,253_ = 1.67, *P* = 0.008, Year*TimesInvited: F_11,253_ = 4.64, *P* < 0.001, Journal*TimesInvited: F5,253 = 2.61, P = 0.03

Lastly, Albert et al. [7] highlight “discrepancies” that they cannot reconcile for reviewer responses at *Functional Ecology* between their re-analysis of data from Fox et al. ([10]; data available at Dryad, datadryad.com; [11]) and their re-analysis of data from Petchey et al. ([8]; also available at Dryad [9]). They find good agreement between the two datasets from 2007 to 2010 but not prior to 2007 (see Figure 4 in [7]). There are at least three factors producing the observed discrepancies. (1) The Fox et al. [10, 11] data include only standard research papers, whereas the Petchey et al. [8, 9] data include editorials, reviews, and other non-standard papers. (2) The Fox et al. [10, 11] study treated invitation responses for which the invitee did not respond to the review request as missing data (because “no response” was not consistently recorded until 2007). This did not impact the results of Fox et al. [10] because that analysis examined each step of the reviewer recruitment process separately and excluded pre-2007 data from analysis of variables that could be affected by the missing data. Accounting for reviewer non-responses (as done here in Fig. 1) leads to substantially improved agreement between the two analyses. (3) Unbeknown to Albert et al. [7], the dataset of Petchey et al. [8, 9] double counts review invitations for some journals and some years if a revision of the manuscript is submitted (this is at least in part because *ScholarOne Manuscripts* [previously *Manuscript Central*] automatically listed individuals who reviewed an original version as reviewers on a submitted revision and counted them as invited in the report of invited reviewers whether they were invited or not). This double counting had little effect on most results presented in Albert et al. [7], but it did inflate the estimated number of invitations sent to each reviewer in the early years of their dataset for journals that had enabled this automatic reviewer selection on revisions (see Fig. 2 for corrected numbers without the double counting). Importantly, none of these dataset problems influence the main conclusions of Albert et al. [7], though analysis of the expanded dataset presented in this commentary shows that editor success in recruiting reviewers has declined more substantially, at least at four of the journals examined here, than Albert et al. [7]) estimated.

In summary, we find that the proportion of invitees who submit a review has been decreasing slowly but steadily for four of the six journals examined here and that the cumulative effect over 13 years has been quite substantial for these journals. Why two of these journals (*Evolution* and *Methods in Ecology and Evolution*), plus a third journal examined by Albert et al. [7] (*Molecular Ecology*), have not experienced a similar decline, are unclear. It could be due to differences in editorial practices at these journals; e.g., although editors select the reviewers to be invited, three of the journals with the most significant declines in the proportion of reviewers agreeing to review—*Functional Ecology, Journal of Ecology*, and *Journal of Applied* Ecology (but not *Journal of Animal Ecology*)—have editorial assistants who contact prospective reviewers on behalf of editors, whereas editors themselves send the reviewer invitations for both journals that showed no significant decline, *Methods in Ecology and Evolution* and *Evolution*. Alternatively, it could be due to differences in the communities they serve—those that have experienced consistent reviewer response rates over time publish more evolutionarily and genetically focused research, whereas those that have shown substantial declines are more ecological in scope. We also find, like Vines et al. [6] and Albert et al. [7], that the average number of invitations being sent to prospective reviewers has not changed much over the 13 years we examine, at least within these journals, suggesting that reviewer fatigue is not the primary reason for the decline in the proportion of invitees who agree to review. We do see evidence that reviewer fatigue may occur at the per-individual level; individuals who receive the most invitations are the most likely to decline the invitation, but too few individuals receive enough invitations (at least within journals) for this to be a primary explanation for the declining proportion of individuals who agree to review.

## Conclusions

Taken together, the data presented here and in Albert et al. [7] suggest that whatever is driving the decrease in reviewer agreement rate is external to the peer review system: even though submissions have increased, review requests per person have not (Fig. 2), at least not within journals. It may be that the rising number of journals or the increase in journal rejection rates (at least at top tier journals, causing papers to cascade among journals) are increasing per-individual reviewing workload, but these should be functionally similar to individual journals receiving more submissions. Moreover, recent modelling work by Kovanis et al. [12] showed that there is normally sufficient capacity within the reviewer pool to cope with increased submissions. One potential cause of the decline in agreement rate is growing demands on researchers’ time from other areas, such as administration and grant writing. The latter is supported by the steady decline in application success rates at, e.g., the National Science Foundation [https://www.nsf.gov/nsb/publications/2014/nsb1432.pdf]. Another possible cause is the apparent growing dissatisfaction with commercial publishers. It would therefore be interesting to repeat this analysis for a non-profit open access publisher such as the Public Library of Science (PLoS).
